# Analysis of regional economic development based on land use and land cover change information derived from Landsat imagery

**DOI:** 10.1038/s41598-020-69716-2

**Published:** 2020-07-29

**Authors:** Chao Chen, Xinyue He, Zhisong Liu, Weiwei Sun, Heng Dong, Yanli Chu

**Affiliations:** 1 Marine Science and Technology College, Zhejiang Ocean University, Zhoushan, 316022 China; 2 College of Mathematics, Physics and Information, Zhejiang Ocean University, Zhoushan, 316022 China; 3 Department of Geography and Spatial Information Techniques, Ningbo University, Ningbo, 315211 China; 4 School of Resources and Environmental Engineering, Wuhan University of Technology, Wuhan, 430070 China; 5 School of Economics and Management, Zhejiang Ocean University, Zhoushan, 316022 Zhejiang China

**Keywords:** Engineering, Environmental sciences, Environmental social sciences

## Abstract

The monitoring of economic activities is of great significance for understanding regional economic development level and policymaking. As the carrier of economic activities, land resource is an indispensable production factor of economic development, and economic growth leads to increased demand for land as well as changes in land utilization form. As an important means of earth observation, remote-sensing technology can obtain the information of land use and land cover change (LUCC) related to economic activities. This study proposes a method for analysing regional economic situations based on remote-sensing technology, from which LUCC information extraction, sensitivity factor selection, model construction and accuracy evaluation were implemented. This approach was validated with experiments in Zhoushan City, China. The results show that the economic statistical index is most sensitive to the construction land area, and the average correlation coefficient between the actual data and the predicted data is 0.949, and the average of mean relative error is 14.21%. Therefore, this paper suggests that LUCC could be utilised as an explanatory indicator for estimating economic development at the regional level, and the potential applications of remotely-sensed image in economic activity monitoring are worth pursuing.

## Introduction

The monitoring of economic activities is of great significance for the understanding of economic situations and the support of policymaking concerned with sustainable development and management^1^. Considering that economic activities are cumulatively changing the surface of the Earth, data that reveal the Earth’s surface changes can therefore enable the capability of frequent and large-scale observations of economic activity, which could substantially improve understanding of the actual economic situation and its trend prediction. Traditional data collection methods such as mapping and ground surveying are time-consuming and costly^2^. Additionally, the information is not updated frequently and is difficult to access^3,4^. A powerful implementation of economic activity monitoring research is remotely-sensed image, which provide an up-to-date and realistic presentation of the Earth’s surface. Remote-sensing technology is an effective means of observing surface changes on the Earth due to its fast and wide-range imaging capability^5–10^. Since the 1970s, terrestrial Earth observation data have been continuously collected in various spectral, spatial and temporal resolutions^11,12^. In recent decades, the accessibility, quality and scope of these data have been continuously improving, making it a fundamental information source in the study of pattern change and visualization of the Earth’s surface as well as important data in the research of human activities monitoring^13,14^.

Having the capability to detect low levels of visible and near-infrared (VNIR) radiance at night, the Defense Meteorological Satellite Program-Operational Linescan System (DMSP-OLS) night-time light (NTL) data provided a new scope for measuring human economic activities^15–20^. These NTL data are free and feature a wide spatial coverage from − 180° to 180° longitude and − 65° to 75° latitude, thus greatly enhancing NTL application research^21^. As an objective reflection of human activities, NTL data provide a cost-effective and spatially consistent means for monitoring economic activities^22–25^. The initial purpose of the DMSP/OLS, however, was to observe the clouds illuminated by moonlight, and the night-time light imagery was a by-product of the data under cloud-free conditions^26–30^. Consequently, there remain some limitations when using NTL data: (1) Underestimation of economic activities that emit less or no additional night-time light, and in the potentially serious measurement errors of gross domestic product (GDP) growth, particularly in developing and emerging economies, the growth is more likely to be underestimated^31–33^. (2) As the most important NTL data, DMSP-OLS data are provided by multiple DMSP satellites, and the fact that NTL data from different satellites in different years cannot be directly compared due to lack of onboard calibration is likely to be the main obstacle to a time-series analysis^34–37^. (3) The DMSP-OLS NTL data provided by the NGDC have a geographic grid resolution of 30 arc seconds and a grid cell size that is approximately 0.86 km^2^ at the equator^16^. Therefore, the probability of multiple ground objects belonging to the same pixel is large, which affects the accuracy of economic activity evaluation^38^.

Generally, satellite remote-sensing missions are originally designed to monitor the physical environment of the Earth, for which the mapping of LUCC information is one of the most important applications^39,40^. LUCC information is usually associated with and mainly driven by socioeconomic factors and is also a direct reflection of economic activities^41–43^. It is well documented that the relationship between economic growth and LUCC information is not a one-way effect, but rather a complex relationship of interactions^44,45^. On the one hand, economic activities have profoundly changed the surface morphology of the Earth. Moreover, with economic development and population increase, land use changes have accelerated sharply, and the land cover pattern changes have become more and more significant. On the other hand, the variation process of land use and land cover has significant impacts on economy. As the foundation for economic activities, land is an indispensable production factor for economic development, and the input of land resources plays an important role in promoting economic growth^34,46,47^. Thus, in the coordinated process of LUCC information and economic development, changes in economic activity intensity can be reflected through LUCC information. Remotely-sensed image can intuitively and comprehensively reflect the dynamics of land use and land cover, and the types, quantities and locations of LUCC information can be obtained via classification technology from remotely-sensed image. Normal multispectral optical satellite data with various spatial, temporal and spectral resolutions have been extensively applied in investigations of LUCC information and its driving mechanism of socioeconomic factors^44,48^. However, economic indicators for assessing economic development have rarely been connected to LUCC data in its estimation in time series. The LUCC information derived from remotely-sensed images such as those from Landsat and MODIS is spatial and temporal, and this information does not require further postprocessing for comparison with NTL data^49,50^. Moreover, with the launch of satellites such as QuickBird, IKONOS, GeoEye, WorldView, SPOT 6/7, and GF-1/2 with higher spatial resolution, opportunities are provided for the global production of LUCC at the scale of 10 m or even meters^51–54^.

This study proposes a method to analyse regional economic situations based on the LUCC dynamics derived from remotely-sensed image. The main steps are as follows. First, multi-temporal remotely-sensed image is used to obtain the LUCC information (types and their areas) over a long period of time in the study area. Second, correlation analysis is applied to select the optimal indicators of economic situations (described by various economic statistical indices) from the LUCC indicators. Then, regression analysis is applied in order to model the socioeconomic indicators. Finally, the method accuracy and model applicability are evaluated. The objectives of this study were to quantify the relationship between LUCC area and several socioeconomic statistics over time and to test the capability of LUCC to estimate regional economic development. This study will result in significant understanding of the regional economic development as well as assessing the data accuracy of social survey activities.

## Methodology

**Figure 1:**
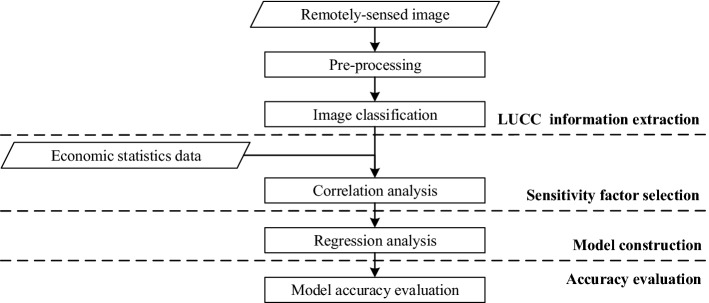
Flow chart of the proposed method.

The interrelationship between LUCC information and economic development is the basis of the proposed method, which attempted to reflect economic development using remotely-sensed images. The overall workflow (Fig. 1) was divided into 4 steps: (1) LUCC information extraction (in “LUCC information extraction” section), (2) sensitivity factor selection (in “Sensitivity factor selection” section), (3) model construction (in “Model construction” section) and (4) accuracy evaluation (in “Accuracy evaluation” section).

The software packages used for this study were environment for visualizing images (ENVI) for image processing, ArcGIS and MATLAB were used for analysing and presenting the results, and statistical product and service solutions (SPSS) was used for statistical analysis.

### LUCC information extraction

The LUCC information is obtained from remotely-sensed time series data. First, pre-processing was performed on the remotely-sensed image, including radiometric calibration, atmospheric correction and image cropping. The digital numbers in the raw data were converted to the top of atmosphere (TOA) reflectance by physical means via calibration parameters provided by Calibration Parameter Files (CPF), and the influence of atmospheric scattering and absorption were reduced by using Fast line-of-sight atmospheric analysis of spectral hypercubes (FLAASH) in ENVI. Then, based on features such as the spectral and spatial resolutions of the image, the training samples were selected and the maximum likelihood classification (MLC) algorithm in supervised classification was carried out in order to obtain the land use types and their associated areal coverage within the study area. Considering the visual separability of different ground objects, the training samples were selected for six classes such as construction land, water, bare land, forest, tidal flat, crop land, and the training samples were divided into two parts, two-thirds for classification, and one-thirds for accuracy assessment. The reliable accuracy of classification was performed using overall accuracy and Kappa coefficient computed, and the overall accuracy is a measure of how well the classified pixels match the ground truth data while the Kappa coefficient measures how well the classification in question would compare to a chance arrangement of pixels to each land cover class. Finally, linear interpolation was performed by Eq. (1) on data with missing years, since remotely-sensed image may not cover all years.

1 $$Data_{INI + i} = Data_{INI} + \frac{{Data_{TER} - Data_{INI} }}{TER - INI} \times i$$

where *Data*_*INI*+1_ is the data of *i*th year after the initial year *INI*, *Data*_*INI*_ and *Data*_*TER*_ are the data of the initial year *INI* and the termination year *TER*, respectively, and the data of the years between the initial year *INI* and the termination year *TER* is missing.

In the study, the overall accuracy and Kappa coefficient were computed by Eq. (2) using the confusion matrix, which is a square array of numbers set out in rows and columns which express the number of sample units (i.e., pixels, clusters of pixels, or polygons) assigned to a particular category relative to the actual category as verified on the ground^55–57^.

2 $$\left\{ {\begin{array}{*{20}l} {OvAc = \mathop \sum \limits_{i = 1}^{r} {{x_{ii} } \mathord{\left/ {\vphantom {{x_{ii} } N}} \right. \kern-\nulldelimiterspace} N}} \hfill \\ {k_{hat} = \frac{{N\mathop \sum \nolimits_{i = 1}^{r} x_{ii} - \mathop \sum \nolimits_{i = 1}^{r} \left( {x_{i + } x_{ + i} } \right)}}{{N^{2} - \mathop \sum \nolimits_{i = 1}^{r} \left( {x_{i + } x_{ + i} } \right)}}} \hfill \\ \end{array} } \right.$$

where *OvAc* is and *k*_*hat*_ are overall accuracy and Kappa coefficient, respectively, *r* is the number of rows in the matrix (the total number of categories), *x*_*ii*_ is the number of observations in row *i* and column *i* (the total pixels number of corrected classifications in training samples used for accuracy assessment), *x*_*i*+_ and *x*_+*i*_ are the marginal totals of row *i* and column *i*, respectively, and *N* is the total number of observations (the total pixels number of training samples used for accuracy assessment).

### Sensitivity factor selection

The interaction mechanism between economic growth and LUCC information is complex, and eleven economic indices are selected to describe economic status: gross domestic product (GDP), value-added of primary industry (VPI), value-added of secondary industry (VSI), value-added of tertiary industry (VTI), per capita GDP (PGDP), fixed assets investment (FAI), total tourist income (TTI), gross industrial output value (GIOV), gross agricultural output value (GAOV), gross planting output value (GPOV) and gross forestry output value (GFOV). Therefore, the land category that is most relevant to the economic statistical index must be selected as sensitive factor to construct the model for estimating socioeconomic situations. The correlation coefficients between the economic statistical index and the land use type by Eq. (3) using correlation analysis. For each economic statistical index, the most relevant land use type was selected as the sensitivity factor, i.e., the explanatory variable in the model.

3 $$r_{LUCCm - ESIn} = \frac{{\mathop \sum \nolimits_{i = 1}^{N} \mathop \sum \nolimits_{j = 1}^{N} \left( {x_{LUCCmi} - \overline{{x_{LULCm} }} } \right)\left( {x_{ESInj} - \overline{{x_{ESIn} }} } \right)}}{{\sqrt {\mathop \sum \nolimits_{i = 1}^{N} \left( {x_{LUCCmi} - \overline{{x_{LUCCm} }} } \right)^{2} } \sqrt {\mathop \sum \nolimits_{i = 1}^{N} \left( {x_{ESInj} - \overline{{x_{ESIn} }} } \right)^{2} } }}$$

where $r_{LUCCm - ESIn}$ is the correlation coefficient between land use type *m* (one in construction land, water, bare land, forest, tidal flat and crop land) and economic statistical index *n* (one in GDP, VPI, VSI, VTI, PGDP, FAI, TTI, GIOV, GAOV, GPOV and GFOV), $x_{LUCCmi}$ and $x_{ESInj}$ ${x}_{ESInj}$ are *i*th land use type *m* and *j*th economic statistical index in category *n*, respectively, $\overline{{x_{LULCm} }}$ and $\overline{{x_{ESIn} }}$ are the average of land use type *m* and the average of economic statistical index *n*, respectively, *N* is the total number of land use type *m* or the total number of economic statistical index *n*.

### Model construction

Regression analysis was applied when using the LUCC information to model the economic statistical indices. In order to eliminate heteroscedasticity and clarify the relationship between the LUCC information and the economic statistical indices more accurately, the logarithmically transformation base 10 were performed to change the range and scale of the data. We attempted to construct a single-factor quantitative model in which each economic statistical index is a dependent variable and the area of each land use type is an independent variable. The model is described by Eq. (4):

4 $$I_{economic} = f\left( {L_{landuse} } \right)$$

where $I_{economic}$ is the value of a given economic statistical index, $L_{landuse}$ is the area of the land use type that is selected as the sensitivity factor for this economic statistical index, and *f* is the quantitative model.

In this study, by inspecting scatter plots, a series of comparative statistical regression analyses are conducted, including linear, quadratic-term, power and exponential models. 4 types of simple quantitative models were constructed as shown in Table 1.

**Table 1:** The type and representation of the model.

The type of the model	The representation of the model
Linear model	*y* = *a* + *bx*
Quadratic-term model	*y* = *a* + *bx* + *cx*^*2*^
Power model	*y* = *ax*^*b*^
Exponential model	*y* = *ae*^*bx*^

Where *y* is the economic index of the logarithmic transformed base 10, *x* is the area of land use type of the logarithmic transformed base 10, and *a*, *b* and *c* are coefficients.

### Accuracy evaluation

Accuracy evaluation was performed to validate the model. For the model validation data, the independent variable was inserted into the regression model to obtain the estimated value, which was then compared with the actual value. The relative error (RE) is the ratio of the absolute error to the actual value, which can reflect the deviation of the model prediction from the actual value. The mean relative error (MRE) was used to evaluate the overall accuracy of the models. The formula is as follows:

5 $$MRE = \frac{1}{n}\mathop \sum \limits_{i = 1}^{n} \left| {\frac{{y_{e} - y_{a} }}{{y_{a} }}} \right|$$

where *y*_*e*_ and *y*_*a*_ are the model estimated value and the actual value, respectively, and *n* is the number of the actual value.

## Study area and data

### Study area

**Figure 2:**
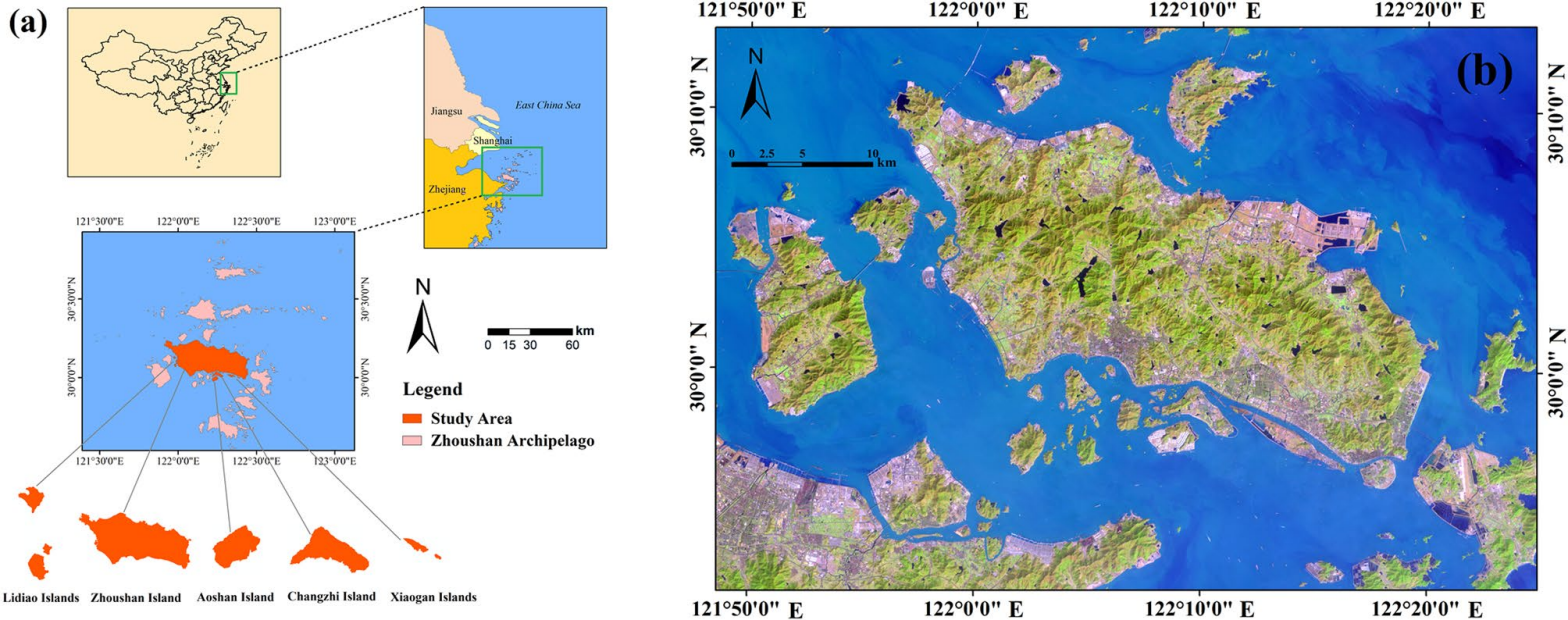
Location map of the study area. (**a**) The geo-location of the study area, (**b**) The remotely-sensed image of the study area is false-color composite of Landsat-8 OLI images that rank band composites in the order of short wave-infrared (SWIR), near-infrared (NIR), and red bands. Map created in ArcMap 10.5 of the Environmental System Resource Institute, Inc. (www.esri.com/software/arcgis/arcgis-for-desktop). Boundaries made with free vector data provided by National Catalogue Service for Geographic Information (https://www.webmap.cn/commres.do?method=dataDownload).

Founded in 1987, Zhoushan is the first prefecture-level city in China that consists of islands; specifically, the Zhoushan Archipelago, which consists of 1390 islands with areas greater than 500 m^2^^58^. Zhoushan City is located on the coast of the East China Sea, west of Hangzhou Bay and north of Shanghai (Fig. 2). It has a total administrative area of approximately 22,200 km^2^, but a land area of only 1140.12 km^2^. Zhoushan has abundant marine economic resources and is well known for marine fishery, tourism, international shipping and shipbuilding industries^58^. In 2011, the Zhoushan Archipelago New District was established. This was the first national strategic-level new district in China with a marine economy theme.

Zhoushan is characterised by hilly landforms, with numerous mountains and hills on the islands. Thus, land that can be effectively used is scarce. For this reason, the land development intensity of the islands varies greatly and the core zones of the city are located on the islands with larger areas. Zhoushan Island is the largest in Zhoushan City and also its economic and political center, and the area of Zhoushan Island is 502.65 km^2^, its east–west length is 44 km and its north–south width is 18 km^59^. The study area presented in Fig. 2a includes Zhoushan Island, Changzhi Island, Aoshan Island, Xiaogan Islands, and Lidiao Islands. The total land area of study region is 529.38 km^2^. This area is the core zone of Zhoushan and has experienced dramatic LUCC due to the rapid economic growth of the city in recent decades^58^. The original remotely-sensed image of the study area acquired on February 22, 2020 from the Landsat-8 OLI image is presented in Fig. 2b. The image are clear and high quality because of good weather conditions, and the study area includes the ocean, lakes, river, urban areas, wetlands, forest, and other features.

### Data

The data used in this study can be classified into 2 groups: remotely-sensed image and regional statistics of the study area. The remotely-sensed image for a particular day of a given year were used to derive the annual LUCC dynamics of the study area using classification technology of remotely-sensed image. The regional statistics were used to characterise the regional economic development situations for each calendar year.

#### Remotely-sensed image

We attempted to determine the LUCC information of the study area from remotely-sensed image since the city was established. Given the limitations and constraints in the acquisition and selection of proper images, Landsat satellite images were used to derive the LUCC information in the study area. Landsat is a series of terrestrial satellites launched by NASA. Since 1972, 8 satellites have been launched, of which the Landsat 6 satellite failed to transmit. At present, the Landsat satellites have been continuously observing the Earth for more than 40 years and have accumulated large-scale, long-term remotely-sensed image, which are widely used in Earth observation research^21,32^. Landsat satellites have basically the same observation conditions and 16- or 18-day re-entry cycles. In addition, the thematic mapper (TM), enhanced thematic mapper (ETM+), operational land imager (OLI) on Landsat satellite are the multi-spectral sensor with spatial resolution of 30 m (except for several spectral bands), which is better than the NTL data.

Considering the availability of cloud-free spatial coverage and the consistency of the annual acquisition date, 11 Landsat TM or OLI images spanning 32 years (1984–2016) were used to obtain the multi-temporal LUCC information of the study area (Table 2). The collected images were provided by the US Geological Survey (USGS) (https://glovis.usgs.gov/) and the Geospatial Data Cloud Platform of the Chinese Academy of Sciences Computer Network Information Center (https://www.gscloud.cn). The image format is GeoTIFF and the coordinate system is World Geodetic System 1984 (WGS84) projected by Universal Transverse Mercator (UTM) Projection. For the Landsat TM images, only the 6 reflective bands with 30-m spatial resolution were used for further data analysis, while the thermal infrared (TIR) band with a coarse spatial resolution of 120 m was excluded. For OLI images, the Pan band and Cirrus band were excluded, while the other 7 bands with 30-m spatial resolution were used.

**Table 2:** Information of landsat imagery used in the research.

Satellite	Sensor	Acquire date	Path/Row
Landsat 5	TM	April, 1984	118/39
Landsat 5	TM	June, 1990	118/39
Landsat 5	TM	July, 1995	118/39
Landsat 5	TM	April, 1999	118/39
Landsat 5	TM	June, 2005	118/39
Landsat 5	TM	July, 2007	118/39
Landsat 5	TM	July, 2009	118/39
Landsat 5	TM	May, 2011	118/39
Landsat 8	OLI	July, 2013	118/39
Landsat 8	OLI	August, 2015	118/39
Landsat 8	OLI	May, 2016	118/39

#### Socioeconomic dataset

In general, GDP is the most common economic indicator. In this study, we extended the selection of indicators to include those that are, in theory, closely related to LUCC information. We assembled a city-level statistical dataset spanning 32 years (1984–2016) from the statistical yearbook of Zhoushan City, gross domestic product (GDP), value-added of primary industry (VPI), value-added of secondary industry (VSI), value-added of tertiary industry (VTI), per capita GDP (PGDP), fixed assets investment (FAI), total tourist income (TTI), gross industrial output value, (GIOV), gross agricultural output value (GAOV), gross planting output value (GPOV) and gross forestry output value (GFOV). GDP is a monetary measure of the market value of all the final goods and services produced in a specific period, PGDP refers to the per capita GDP, the primary industry (PI) refers to agriculture, forestry, animal husbandry, and fishery (excluding the service industry in agriculture, forestry, animal husbandry, and fishery), the secondary industry (SI) refers to mining (excluding mining auxiliary activities), manufacturing (excluding metal products, machinery and equipment repair), electricity, heat, gas and water production and supply, and construction, the tertiary industry (TI) is the service industry, which mainly includes transportation, communications, commerce, catering, finance, education, and public services, FAI measures the change in the total spending on non-rural capital investments such as factories, roads, power grids, and property in Chines, the TTI refers to the total monetary income obtained by the destination country or region in a certain period from providing tourism products, purchasing goods, and other services to tourists at home and abroad, the GIOV refers to the total result of industrial production activities of an industrial enterprise (unit) in a certain period, which is the total value of industrial final products and industrial labor services provided in money, the GAOV is the total amount of all agricultural, forestry, animal husbandry, and fishery products expressed in monetary form in a certain period (usually 1 year), the GPOV refers to the total amount of plantation and agricultural products expressed in monetary form in a certain period (usually 1 year), and the GFOV refers to the total amount of fishery expressed in monetary form in a certain period (usually 1 year)^13,16,60–63^. The details of these indicators are listed in Table 3. For the PGDP, from 1984 to 2000 it was calculated using the registered population, and from 2000 to 2016 it was calculated using the resident population. In this study, PGDP is in units of yuan (CNY), while the other economic statistics are in units of 10^5^ CNY.

**Table 3:** Socioeconomic dataset of Zhoushan.

Economic statistical index	Abbreviation	Units	Date
Gross domestic product	GDP	10^5^ CNY	1984–2016
Value-added of primary industry	VPI	10^5^ CNY	1984–2016
Value-added of secondary industry	VSI	10^5^ CNY	1984–2016
Value-added of tertiary industry	VTI	10^5^ CNY	1984–2016
Per capita GDP	PGDP	CNY	1984–2016
Fixed assets investment	FAI	10^5^ CNY	1984–2016
Total tourist income	TTI	10^5^ CNY	1984–2016
Gross industrial output value	GIOV	10^5^ CNY	1984–2016
Gross agricultural output value	GAOV	10^5^ CNY	1984–2016
Gross planting output value	GPOV	10^5^ CNY	1984–2016
Gross forestry output value	GFOV	10^5^ CNY	1984–2016

## Experimental results and analyses

### LUCC information extraction

The Landsat satellite images were pre-processed using several procedures, i.e., radiometric calibration, atmospheric correction and image cropping. In classification system construction, the spatial resolution, spectral resolution of remotely-sensed image and the features of ground objects in the study area need to be considered comprehensively. The study area has a complex landscape, with hills in the centers of the islands, making the spatial distribution of the objects discrete and resulting in mixed pixels at the spatial resolution of the images. Due to the significant spectral confusion, we grouped several categories together; specifically, grassland was grouped into cropland; aquaculture and brine pan were grouped into tidal flat. Finally, the LUCC information in the study area was divided into 6 categories: (1) construction land, (2) forest, (3) water, (4) bare land, (5) cropland and (6) tidal flat.

Maximum likelihood estimation was applied to the supervised classification of pre-processed Landsat images. The images were visually enhanced using linear contrast stretching and different band combinations to help select training samples. The classification results were modified and corrected in order to eliminate obvious errors. Accuracy assessment was performed for each classification result, and the samples were chosen from the repository of Google Earth historical images. The average value of the overall classification accuracy was 84.05%, and the average value of the Kappa coefficient was 0.80, as shown in Table 4.

**Table 4:** Classification accuracy assessment.

Year	Overall accuracy (%)	Kappa coefficient
1984	84.92	0.81
1990	76.58	0.71
1995	82.48	0.76
1999	87.05	0.84
2005	87.11	0.82
2007	82.73	0.78
2009	88.73	0.81
2011	83.80	0.81
2013	83.18	0.80
2015	84.44	0.81
2016	83.50	0.80
Average	84.05	0.80

**Figure 3:**
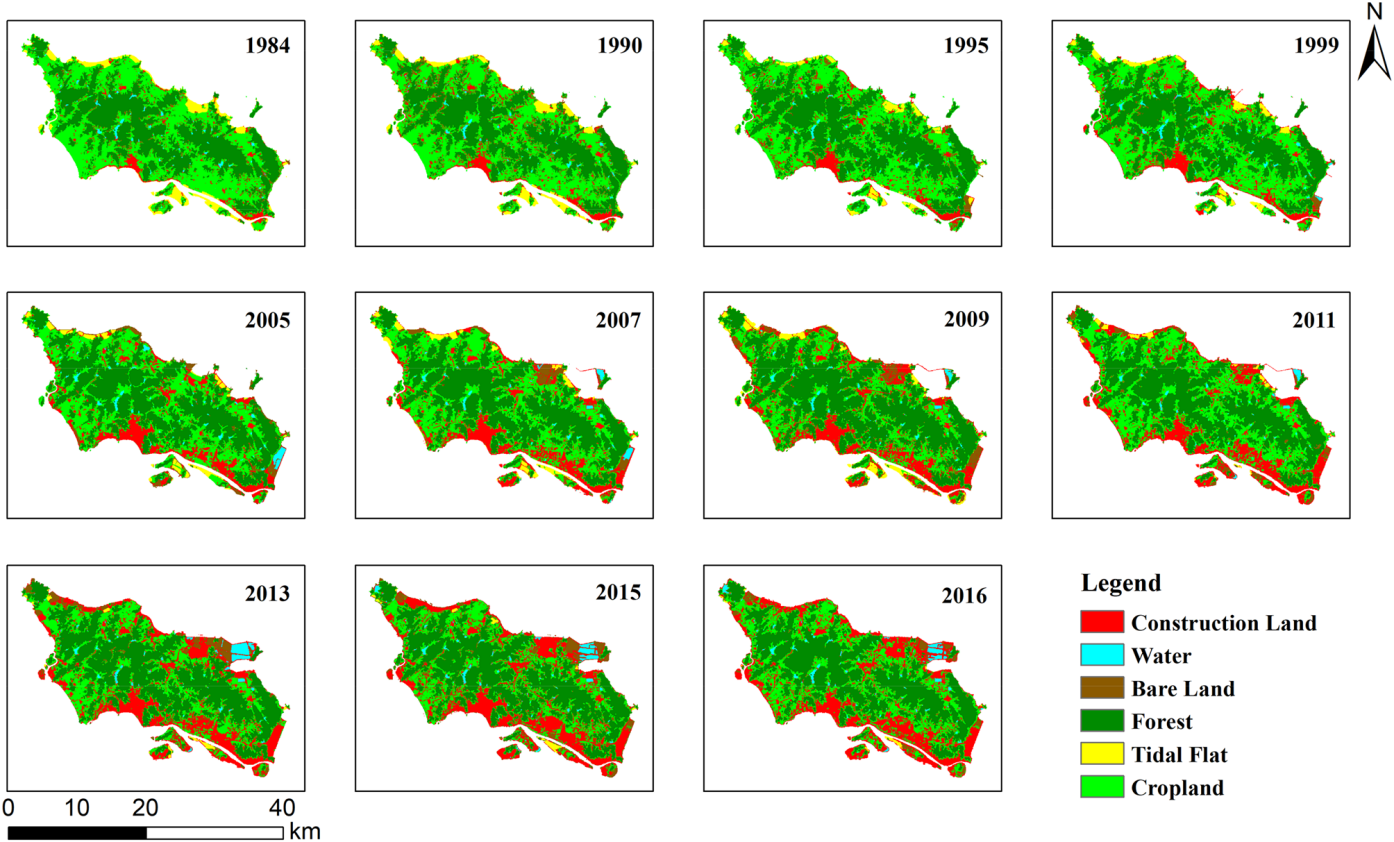
Classification maps of the 11 years examined in this study. Map created in ArcMap 10.5 of the Environmental System Resource Institute, Inc. (www.esri.com/software/arcgis/arcgis-for-desktop).

The final classification maps for each of the 11 years are shown in Fig. 3, and the statistics of each category from 1984 to 2016 are listed in Table 5, and based on the classification results, the areas of land use types for missing years were obtained by the linear interpolation. In the Table 5, the bold and the italics represent the original data and the interpolated data, respectively.

**Table 5:** Areas of land use types.

No.	Year	Construction land	Water	Bare land	Forest	Tidal flat	Cropland
**1**	**1984**	**19.57**	**5.62**	**19.84**	**210.72**	**29.35**	**220.70**
*2*	*1985*	*20.79*	*5.52*	*23.61*	*208.97*	*28.64*	*217.98*
*3*	*1986*	*22.01*	*5.41*	*27.38*	*207.22*	*27.92*	*215.26*
*4*	*1987*	*23.23*	*5.31*	*31.16*	*205.48*	*27.20*	*212.55*
*5*	*1988*	*24.45*	*5.21*	*34.93*	*203.73*	*26.49*	*209.83*
*6*	*1989*	*25.67*	*5.10*	*38.70*	*201.99*	*25.77*	*207.11*
**7**	**1990**	**26.89**	**5.00**	**42.47**	**200.24**	**25.06**	**204.39**
*8*	*1991*	*28.75*	*4.79*	*38.64*	*204.72*	*23.38*	*204.22*
*9*	*1992*	*30.61*	*4.58*	*34.81*	*209.20*	*21.70*	*204.05*
*10*	*1993*	*32.47*	*4.37*	*30.97*	*213.69*	*20.01*	*203.89*
*11*	*1994*	*34.33*	*4.16*	*27.14*	*218.17*	*18.33*	*203.72*
**12**	**1995**	**36.19**	**3.95**	**23.31**	**222.65**	**16.65**	**203.55**
*13*	*1996*	*39.66*	*4.78*	*19.54*	*221.43*	*16.43*	*204.49*
*14*	*1997*	*43.12*	*5.61*	*15.77*	*220.21*	*16.21*	*205.43*
*15*	*1998*	*46.58*	*6.44*	*12.00*	*218.99*	*15.99*	*206.37*
**16**	**1999**	**50.05**	**7.27**	**8.23**	**217.77**	**15.77**	**207.31**
*17*	*2000*	*52.20*	*7.63*	*11.66*	*219.98*	*15.15*	*201.55*
*18*	*2001*	*54.34*	*8.00*	*15.10*	*222.18*	*14.53*	*195.80*
*19*	*2002*	*56.49*	*8.36*	*18.53*	*224.39*	*13.91*	*190.04*
*20*	*2003*	*58.63*	*8.72*	*21.96*	*226.60*	*13.30*	*184.28*
*21*	*2004*	*60.78*	*9.08*	*25.39*	*228.80*	*12.68*	*178.53*
**22**	**2005**	**62.92**	**9.45**	**28.83**	**231.01**	**12.06**	**172.77**
23	*2006*	*74.57*	*8.61*	*21.46*	*231.27*	*13.62*	*172.34*
**24**	**2007**	**86.22**	**7.77**	**14.10**	**231.54**	**15.17**	**171.91**
25	*2008*	*89.80*	*7.08*	*18.19*	*244.28*	*16.53*	*154.83*
**26**	**2009**	**93.37**	**6.38**	**22.28**	**257.02**	**17.89**	**137.75**
27	*2010*	*96.05*	*6.20*	*19.09*	*241.92*	*11.97*	*157.93*
**28**	**2011**	**98.72**	**6.03**	**15.91**	**226.82**	**6.05**	**178.11**
29	*2012*	*107.55*	*9.18*	*20.46*	*234.49*	*4.81*	*160.38*
**30**	**2013**	**116.38**	**12.32**	**25.02**	**242.15**	**3.56**	**142.66**
31	*2014*	*119.09*	*12.06*	*34.36*	*236.17*	*3.66*	*138.15*
**32**	**2015**	**121.80**	**11.80**	**43.69**	**230.19**	**3.76**	**133.65**
**33**	**2016**	**131.51**	**11.18**	**20.29**	**226.78**	**1.95**	**159.10**

Note: In this table, the unit of area is km^2^.

The study area has obvious characteristics of LUCC information over the past few decades. First, construction land has increased more than fivefold, while tidal flats and cultivated land/grassland have decreased significantly. The construction land area continually increased over the 32-year study period, from 19.57 to 131.51 km^2^, an increase of 111.94 km^2^ and an increase ratio of 572.03%. Conversely, the tidal flat area decreased by 27.40 km^2^, from 29.35 to 1.95 km^2^, translating to a decrease ratio of 93.35%. The area of cultivated land/grassland decreased by 61.60 km^2^, from 220.70 to 159.10 km^2^, or by 27.91%. Meanwhile, forest land changed relatively little in ratio and area. The forest area increased by 16.06 km^2^, from 210.72 to 226.78 km^2^, translating to an increase of 7.62%, while the water area increased by 5.56 km^2^, from 5.62 km^2^ to 11.18 km^2^, an increase ratio of 98.80%. Finally, the area of bare land fluctuated greatly, but the overall change was not obvious, exhibiting a net increase of 0.45 km^2^ during the study period, from 19.84 to 20.29 km^2^, or 2.26%.

### Sensitivity factor selection

Given that LUCC information is a complex process driven by socioeconomic factors, the primary challenge for estimating economic situations using LUCC information is to determine the association between the area of land use type and the economic statistics. Pearson correlation analysis was applied to qualitatively examine the statistical dependence between area of land use type and economic statistics across the study period. The Pearson correlation coefficient (ranging from − 1 to 1) was used to indicate the sensitivity level of land use type versus economic indices. In addition, the statistical significance level was tested using two-tailed t-statistics.

In order to make LUCC information consistent with economic statistics, linear interpolation was performed on missing-year data. Consequently, we obtained 33 sets of raw data consisting of the LUCC information and economic statistics for every year from 1984 to 2016. This raw dataset was then logarithmically transformed (in base 10, described as lg) in order to eliminate the intrinsic exponential growth trend of economic indicators and to make the data more consistent with the normal distribution, which is the assumption of Pearson correlation analysis. We extracted one-thirds of the dataset at equal intervals for model validation, with the remaining data used for modelling. The correlation coefficients between each economic index and the area of each land cover type for the data used to construct the model are listed in Table 6. These results reveal that all of the economic indices are positively correlated with construction land, forest and water, and negatively correlated with cropland, bare land and tidal flat. The relevance varies, but it is obvious that each economic index is significantly correlated with construction land area; indeed, the coefficients have the highest values among all the land use types. It is apparent that among all of these land use type, the change of construction land is the best explanatory variable for revealing the trend of economic development in the study area. Thus, construction land was selected as the sensitivity factor for the single-factor quantitative model.

**Table 6:** Correlation coefficients between LUCC information and economic statistical index.

	GDP	VPI	VSI	VTI	PGDP	FAI
Construction land	0.996**	0.976**	0.991**	0.995**	0.995**	0.991**
Forest	0.890	0.856**	0.904**	0.892**	0.893**	0.890**
Water	0.731**	0.723**	0.717**	0.736**	0.732**	0.727**
Crop land	− 0.898**	− 0.845**	− 0.907**	− 0.892**	− 0.898**	− 0.912**
Bare land	− 0.306	− 0.354	− 0.289	− 0.315	− 0.309	− 0.260
Tidal flat	− 0.850**	− 0.867**	− 0.817**	− 0.844**	− 0.847**	− 0.864**

	TTI	GIOV	GAOV	GPOV	GFOV	
Construction land	0.984**	0.990**	0.955**	0.932**	0.693**	
Forest	0.892**	0.907**	0.841**	0.856**	0.632**	
Water	0.718**	0.717**	0.674**	0.619**	0.315	
Crop land	− 0.854**	− 0.905**	− 0.806**	− 0.768**	− 0.525*	
Bare land	− 0.368	− 0.290	− 0.354	− 0.421	− 0.302	
Tidal flat	− 0.829**	− 0.815**	− 0.837**	− 0.792**	− 0.595**	

In the table, * indicates the significance level of 0.05, ** indicates the significance level of 0.01, and the bold font indicates the highest relevance.

### Model construction

**Figure 4:**
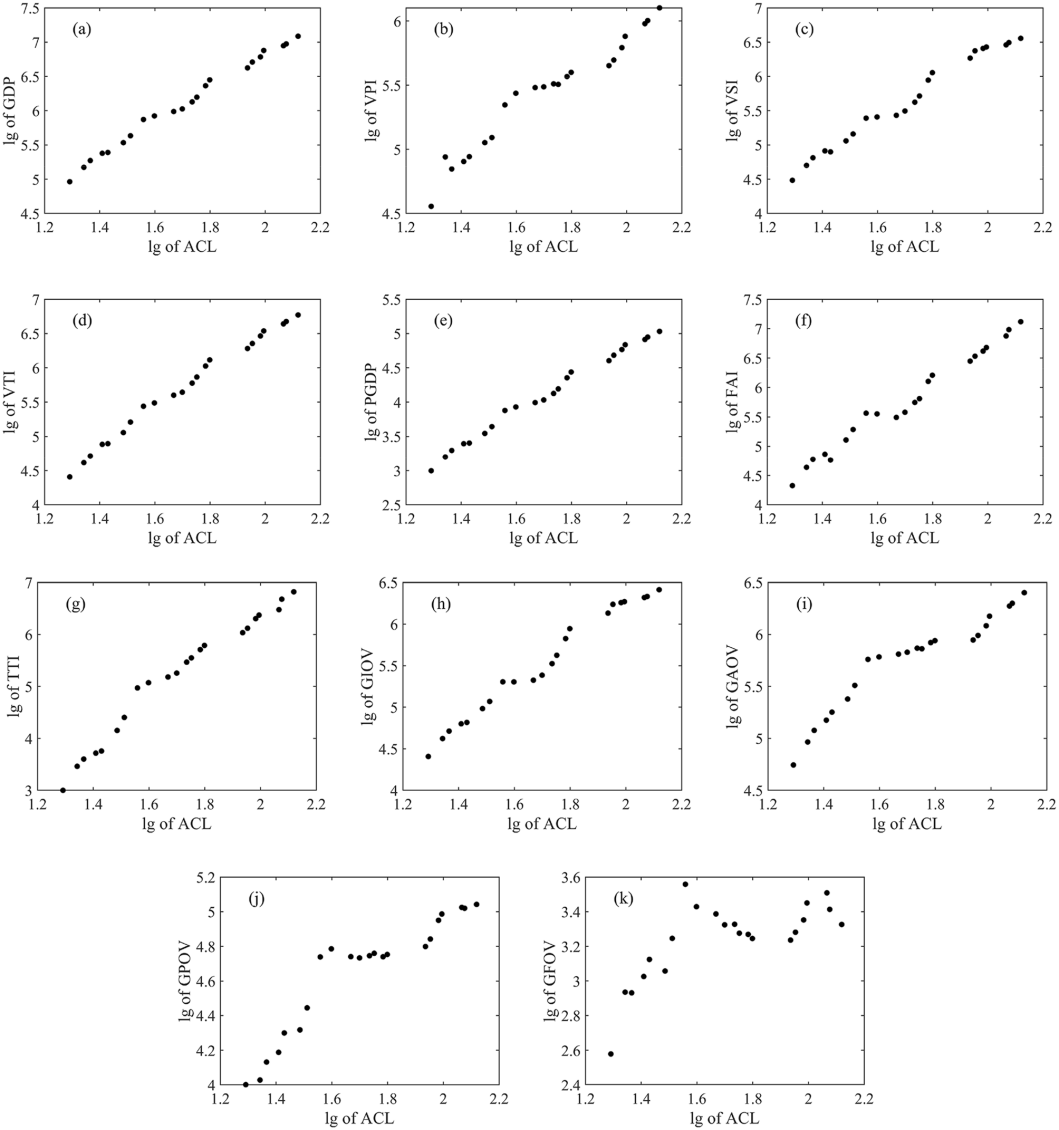
Scatter plots of lg of economic index versus lg of area of construction land (ACL): (**a**) GDP, (**b**) VPI, (**c**) VSI, (**d**) VTI, (**e**) PGDP, (**f**) FAI, (**g**) TTI, (**h**) GIOV, (**i**) GAOV, (**j**) GPOV, (**k**) GFOV. Figures created in MATLAB R2018a of the MathWorks, Inc. (www.mathworks.com).

In order to reduce the impact of large variations in economic index values in time series and to improve the accuracy of regression analysis, a lg–lg regression model was used to estimate economic indices. Taking the lg of the economic index as the dependent variable and the lg of area of construction land (ACL) as the independent variable, the lg–lg scatter plots are presented in Fig. 4.

The coefficients for the lg–lg regression model were estimated by the data used to construct the model. The specific information is shown in Tables 7, 8, 9 and 10; all of the models are significant, with *p* < 0.01.

**Table 7:** Fitting results of the linear model.

Dependent variable (*y*)	Independent variable (*x*)	Model	R^2^
lg (GDP)	lg (ACL)	*y* = 1.874 + 2.478*x*	0.991
lg (VPI)	lg (ACL)	*y* = 2.741 + 1.573*x*	0.952
lg (VSI)	lg (ACL)	*y* = 1.313 + 2.535*x*	0.982
lg (VTI)	lg (ACL)	y = 0.946 + 2.787*x*	0.989
lg (PGDP)	lg (ACL)	y = 0.017 + 2.392*x*	0.991
lg (FAI)	lg (ACL)	y = 0.404 + 3.146*x*	0.982
lg (TTI)	lg (ACL)	*y* = − 2.229 + 4.338*x*	0.968
lg (GIOV)	lg (ACL)	*y* = 1.356 + 2.444*x*	0.981
lg (GAOV)	lg (ACL)	*y* = 2.894 + 1.661*x*	0.911
lg (GPOV)	lg (ACL)	*y* = 2.607 + 1.191*x*	0.869
lg (GFOV)	lg (ACL)	*y* = 2.222 + 0.597*x*	0.481

In the study, lg represents the logarithmically transformation base 10.

**Table 8:** Fitting results of the quadratic-term model.

Dependent variable (*y*)	Independent variable (*x*)	Model	R^2^
lg (GDP)	lg (ACL)	*y* = 0.469 + 4.158*x* − 0.491*x*^2^	0.993
lg (VPI)	lg (ACL)	*y* = 0.6151 + 4.117*x* − 0.744*x*^2^	0.962
lg (VSI)	lg (ACL)	*y* = − 0.176 + 4.316*x* − 0.521*x*^2^	0.984
lg (VTI)	lg (ACL)	*y* = − 1.715 + 5.970*x* − 0.931*x*^2^	0.995
lg (PGDP)	lg (ACL)	*y* = − 1.501 + 4.208*x* − 0.531*x*^2^	0.993
lg (FAI)	lg (ACL)	*y* = 0.260 + 3.319*x* − 0.051*x*^2^	0.982
lg (TTI)	lg (ACL)	*y* = − 10.622 + 14.378*x* − 2.936*x*^2^	0.989
lg (GIOV)	lg (ACL)	*y* = − 0.404 + 4.549*x* − 0.616*x*^2^	0.984
lg (GAOV)	lg (ACL)	*y* = − 1.918 + 7.417*x* − 1.683*x*^2^	0.956
lg (GPOV)	lg (ACL)	*y* = − 1.617 + 6.243*x* − 1.477*x*^2^	0.934
lg (GFOV)	lg (ACL)	*y* = − 2.823 + 6.631*x* − 1.765*x*^2^	0.683

**Table 9:** Fitting results of the power model.

Dependent variable (*y*)	Independent variable (*x*)	Model	R^2^
lg (GDP)	lg (ACL)	*y* = 4.225*x*^0.693^	0.992
lg (VPI)	lg (ACL)	*y* = 4.170*x*^0.497^	0.956
lg (VSI)	lg (ACL)	*y* = 3.748*x*^0.768^	0.984
lg (VTI)	lg (ACL)	*y* = 3.641*x*^0.842^	0.991
lg (PGDP)	lg (ACL)	*y* = 2.394*x*^1.006^	0.990
lg (FAI)	lg (ACL)	*y* = 3.513*x*^0.931^	0.980
lg (TTI)	lg (ACL)	*y* = 2.277*x*^1.519^	0.955
lg (GIOV)	lg (ACL)	*y* = 3.696*x*^0.756^	0.984
lg (GAOV)	lg (ACL)	*y* = 4.383*x*^0.506^	0.922
lg (GPOV)	lg (ACL)	*y* = 3.658*x*^0.449^	0.888
lg (GFOV)	lg (ACL)	*y* = 2.706*x*^0.340^	0.523

**Table 10:** Fitting results of the exponential model.

Dependent variable (*y*)	Independent variable (*x*)	Model	R^2^
lg (GDP)	lg (ACL)	*y* = 3.016 e^0.410*x*^	0.985
lg (VPI)	lg (ACL)	*y* = 3.281 e^0.293*x*^	0.941
lg (VSI)	lg (ACL)	*y* = 2.580 e^0.454*x*^	0.977
lg (VTI)	lg (ACL)	*y* = 2.423 e^0.497*x*^	0.978
lg (PGDP)	lg (ACL)	*y* = 1.470 e^0.594*x*^	0.980
lg (FAI)	lg (ACL)	*y* = 2.232 e^0.551*x*^	0.975
lg (TTI)	lg (ACL)	*y* = 1.106 e^0.889*x*^	0.928
lg (GIOV)	lg (ACL)	*y* = 2.560 e^0.447*x*^	0.975
lg (GAOV)	lg (ACL)	*y* = 3.448 e^0.296*x*^	0.894
lg (GPOV)	lg (ACL)	*y* = 2.959 e^0.262*x*^	0.856
lg (GFOV)	lg (ACL)	*y* = 2.324 e^0.193*x*^	0.479

### Accuracy evaluation

Model validation data were used to verify the models. The estimated economic indices were derived by the models and then compared to the actual values. The MRE is listed in Table 11, showing that the estimation accuracy varies among the models. For most of the economic indices, including VPI, VTI, PGDP, TTI, GAOV and GPOV, the quadratic-term models have higher precision than other models. For VSI and GIOV, linear models have the highest precision. For GDP and FAI, power models display the best performance in terms of precision. Overall, in spite of the model differences, GDP, VPI, VSI, VTI, PGDP, FAI and GIOV are better estimated than TTI, GAOV, GPOV and GFOV.

**Table 11:** Mean relative errors (MREs) of the models.

Economic indices	Linear models (%)	Quadratic term models (%)	Power models (%)	Exponential models (%)
GDP	7.42	7.91	**6.50**	10.63
VPI	14.92	**14.47**	14.53	15.25
VSI	**14.57**	15.51	14.88	15.96
VTI	11.30	**7.61**	10.87	18.12
PGDP	7.49	**7.38**	7.83	12.91
FAI	16.15	16.35	16.44	**14.95**
TTI	37.88	**17.99**	45.81	63.48
GIOV	**14.60**	15.17	14.91	16.63
GAOV	23.06	**17.45**	21.56	25.94
GPOV	19.88	**12.57**	18.36	21.50
GFOV	35.46	**28.19**	34.04	35.15

In the table, the bold font indicates the minimal MRE.

**Figure 5:**
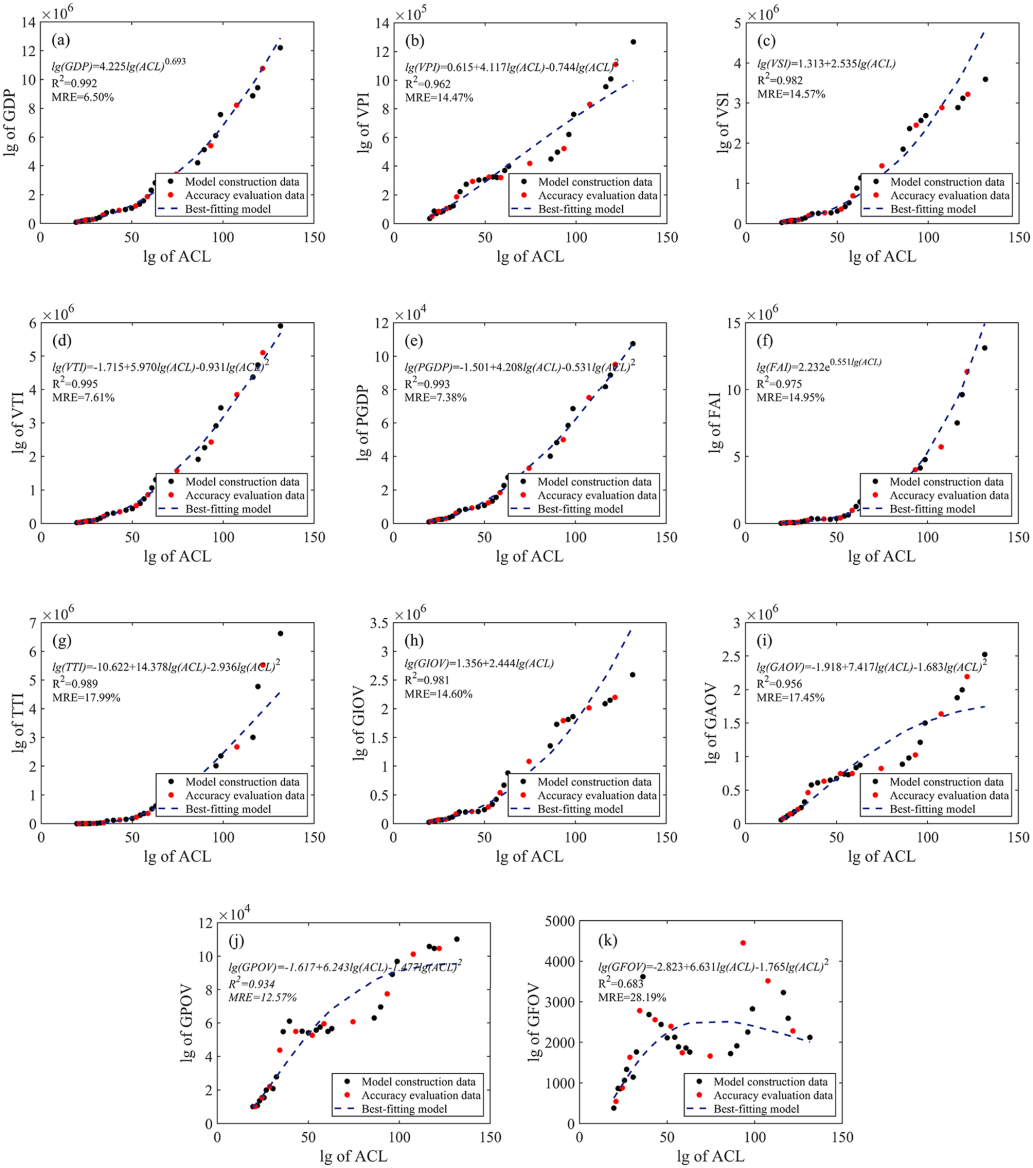
Scatter plots of economic index versus area of construction land and the best-fitting models: (**a**) GDP, (**b**) VPI, (**c**) VSI, (**d**) VTI, (**e**) PGDP, (**f**) FAI, (**g**) TTI, (**h**) GIOV, (**i**) GAOV, (**j**) GPOV, (**k**) GFOV. Figures created in MATLAB R2018a of the MathWorks, Inc. (www.mathworks.com).

The best-fitting models for quantifying the relationship of each economic index were obtained by comparing the MREs of the different models. Among the 4 model types, the model with the lowest MRE was selected as the final model for each economic index. As shown in Table 12 and Fig. 5, the prediction errors of GDP, VTI and PGDP are less than 10%, indicating that these 3 economic indices are quite well estimated by the best-fitting models. For VPI, VSI, FAI, TTI, GIOV, GAOV and GPOV, the errors are also within 20%, which are satisfactory. For GFOV, however, the lowest MRE of 28.19% indicates that the models could not fit it accurately. Overall, the quantitative models could accurately reveal the dynamic changes for most of the economic indicators in this case study.

**Table 12:** Best-fitting models.

Dependent variable (*y*)	Independent variable (*x*)	Model	R^2^	MRE (%)
lg (GDP)	lg (ACL)	*y* = 4.225*x*^0.693^	0.992	6.50
lg (VPI)	lg (ACL)	*y* = 0.615 + 4.117*x* − 0.744*x*^2^	0.962	14.47
lg (VSI)	lg (ACL)	*y* = 1.313 + 2.535*x*	0.982	14.57
lg (VTI)	lg (ACL)	*y* = − 1.715 + 5.970*x* − 0.931*x*^2^	0.995	7.61
lg (PGDP)	lg (ACL)	*y* = − 1.501 + 4.208*x* − 0.531*x*^2^	0.993	7.38
lg (FAI)	lg (ACL)	*y* = 2.232 e^0.551*x*^	0.975	14.95
lg (TTI)	lg (ACL)	*y* = − 10.622 + 14.378*x* − 2.936*x*^2^	0.989	17.99
lg (GIOV)	lg (ACL)	*y* = 1.356 + 2.444*x*	0.981	14.60
lg (GAOV)	lg (ACL)	*y* = − 1.918 + 7.417*x* − 1.683*x*^2^	0.956	17.45
lg (GPOV)	lg (ACL)	*y* = − 1.617 + 6.243*x* − 1.477*x*^2^	0.934	12.57
lg (GFOV)	lg (ACL)	*y* = − 2.823 + 6.631*x* − 1.765*x*^2^	0.683	28.19
Average			0.949	14.21

## Discussion

Remotely-sensed image record changes on the Earth’s surface and thus can be used to represent human activities and to estimate socioeconomic indicators. Traditionally, NTL data are the main remotely-sensed image utilised to estimate socioeconomic situations. Few studies, however, have focused on the quantitative relationship between LUCC information and economic development, which is also a reliable indicator for estimating socioeconomic situations.

This study opens up unique opportunities for the objective, seamless understanding of regional economic development from the perspective of land-use/cover change using remotely-sensed time series data, as well as the correction of economic survey data, both with a high degree of accuracy. The results of the case study in Zhoushan City indicated that LUCC information derived from remotely-sensed image could be indicative of dynamics in economic activity during economic development processes at the city level, as revealed by various quantitative correlations with relevant economic statistics. There is good performance in modelling the economic statistics when the area of construction land is selected as the sensitivity factor. The method proposed in this study still contains some deficiencies and uncertainties, however, as a result of the following factors.

The LUCC information is the key factor affecting the modelling accuracy, since the sensitivity factors selected from LUCC information were the basis for the regressions. The spatial resolution of Landsat imagery was relatively low and the grouping of land use types was made due to the low separability caused by mixed pixels. It is necessary to use high-resolution images to extract more detailed LUCC information, and at the same time, we can use much more suitable classification methods to improve classification accuracy.

1. The spatial matching of remotely-sensed image and statistical data is also one of the influential factors which is currently not perfect and requires further improvement in future studies. Due to an absence of statistical data precisely matching the remotely-sensed image spatially, we had to utilise the LUCC information that only covered the core zone of Zhoushan City when modelling the statistical data at the city level.
2. In the study, only a single factor was included in the modelling, while in reality the correlation analysis showed that several land use types are significantly correlated with economic indices. Thus, additional factors should be included in the modelling, and the analysis of the impacts of different land use types on economic indices is necessary.
3. The existing studies have shown that the interaction between LUCC and economic development displays obvious regional differences. This interaction may be affected by many natural and unnatural factors such as land resource conditions, land policy and economic development stage. Therefore, the reliability of the proposed method needs to be further verified by additional case studies in different areas.

## Conclusions

From the perspective of the interrelationship between LUCC information and economic development, this study proposed a method for analysing regional economic situations using remotely-sensed image to extract LUCC information. Through a case study of Zhoushan, China’s first prefecture-level island city, this research investigated the ability of LUCC information to estimate economic indices. The LUCC information was extracted from Landsat images, taking the area of construction land as the explanatory variable after correlation analysis. Eleven economic indices—GDP, VPI, VSI, VTI, PGDP, FAI, TTI, GIOV, GAOV, GPOV and GFOV—were incorporated in linear, quadratic-term, power and exponential models. The accuracy evaluation revealed that the mean relative errors of the best-fitting models for the 11 economic indices were 6.50%, 14.47%, 14.57%, 7.61%, 7.38%, 14.95%, 17.99%, 14.60%, 17.45%, 12.57% and 28.19%, respectively. In conclusion, the results prove that LUCC information could be used as an explanatory indicator for estimating economic development at the regional level, and the potential applications of remotely-sensed image in the monitoring of economic activities are worth pursuing. In future research, the remotely-sensed image quality still has room for improvement, and more methods could be applied to the classification process.

In this paper, a comprehensive analysis method for analysis of regional economic situation was enriched using remote-sensing technology. The study has some deficiencies, and further work should be conducted regarding (1) more case studies from different regions should be undertaken in order to verify the reliability and applicability of the proposed method; (2) using remotely-sensed image with the higher spatial resolution to obtain more detailed information on land use and cover change, and reduce the impact of mixed pixels on statistics of areas of land use types; (3) using the method such as deep learning to improve the classification accuracy.
